# 3D renal modeling classifies BHD as a ciliopathy, possibly responsive to treatment with PTC124

**DOI:** 10.1186/2046-2530-1-S1-P82

**Published:** 2012-11-16

**Authors:** S Basten, K Haidari, G Slaats, M Steensel van, R Giles

**Affiliations:** 1 University Medical Center Utrecht, the Netherlands; 2 University Medical Center Maastricht, the Netherlands

## 

Ciliopathy is a general term applied to diseases that originate from ciliary dysfunction, which often coincide with renal cyst development. Any syndrome displaying renal cysts, such as folliculin (FLCN) in Birt-Hogg-Dube (BHD) syndrome, might therefore be suspected as a novel ciliopathy. Indeed, FLCN localizes to the primary cilium. To study ciliopathies in detail, we set up a renal 3D culture system using IMCD3 cells that physiologically mimick polarized renal tubuli. SiFlcn was essayed in this system, identifying several affected processes; 1) Reduction of ciliation in vitro and in vivo. 2) Increase in cell volume. 3) Increase of mis-oriented cell divisions. These results suggest Flcn functions in cilia stability/ciliogenesis and planar cell polarity (PCP) regulation. PCP signaling is a form of non-canonical Wnt signaling subjected to ciliary regulation Accordingly, whereas β-catenin is normally present in the axoneme, this is never observed upon Flcn depletion and Wnt signaling appears increased as downstream effector Axin2 is stabilized. This suggests a switch from PCP to canonical signaling. Notably, upon ectopic GFP-FLCN expression, but not the non-functional allele p.L508R, cilia become stabilized, accompanied by an accumulation of both GFP-FLCN and β-catenin in the cilium. The second most common mutant FLCN allele is p.T463X; forced read-through with pharmaceutical agent PTC124, targeting nonsense-mediated decay, was tested and showed a robust response as protein expression appears fully restored. We suggest that BHD regulates ciliary function in a 3D polarized cell assay, and PTC124 might be a simple therapy for the second most frequent allele in BHD carriers.

